# Transfemoral leadless pacemaker implantation after transcatheter tricuspid valve in ring implantation

**DOI:** 10.1007/s00392-023-02186-4

**Published:** 2023-04-29

**Authors:** Abai Turdubaev, Thomas Fink, Thomas Eitz, Guram Imnadze

**Affiliations:** 1 Scientific Research Institute of Heart Surgery and Organ Transplantation, Bishkek, Kyrgyz Republic; 2 Clinic for Electrophysiology, Herz- und Diabeteszentrum NRW, Ruhr-Universität Bochum, Georgstr. 11, Bad Oeynhausen, Germany; 3 Clinic for Thoracic and Cardiovascular Surgery, Herz- und Diabeteszentrum NRW, Ruhr-Universität Bochum, Bad Oeynhausen, Germany

Sirs:

Lauten et al. reported on the first-in-human approach of transcatheter tricuspid valve implantation in 2011 (TTVI). This TTVI method remains well-established today [1]. After TTVI, several options are available for patients who need a permanent pacemaker. A conventional endocardial lead implantation is possible but can lead to severe tricuspid regurgitation (TR). An endocardial lead implantation in the coronary sinus system may serve as a good compromise in many situations, but there are limited data about lead stability in pacemaker-dependent patients. Moreover, both aforementioned strategies could be challenging once the high risk of infectious complications is present.

Another option is an epicardial lead implantation, although it may result in delayed recovery from minimal open-chest surgery and increased postoperative risk. The relatively new option is leadless pacemaker (LP) implantation, which is less invasive than epicardial lead implantation and can reduce postoperative complications compared with traditional intravenous pacemaker system implantation. In this paper, we present the first case of LP implantation through a tricuspid valve after double TTVI using conventional femoral access in a multimorbid high-risk patient.

A 69-year-old patient with a history of dilated cardiomyopathy with a severely reduced ejection fraction (15–20%), permanent atrial fibrillation (since 2018) with a CHADS2-VASc score of 5 points, pulmonary hypertension, cirrhosis of the liver and terminal kidney disease on hemodialysis (via Demers catheter implanted from the left subclavian vein) presented at our Center. The patient has a history of catheter-associated bacteremia with positive blood culture (staphylococcus aureus) for 6 months. His severe tricuspid valve insufficiency had been treated with surgical reconstruction of the tricuspid valve (TV) ring (32 mm) implantation in 2015 together with surgical endocardial cryo-ablation and occlusion of the left atrial appendage (LAA). In 2018, after recurrence of a severe tricuspid valve insufficiency, valve-in-ring implantation of a 29 mm Edwards Sapien S3 (Edwards Lifesciences, Irvine, CA) TAVI valve was performed. Following the first valve deployment, a moderate-to-severe paravalvular leak was seen and another same sized valve-in-valve implantation was undertaken. After the procedure, an only minimal paravalvular leak was present. The patient was also treated for severe mitral valve insufficiency requiring a single mitral clip implantation (PASCAL ACE, Edwards Lifesciences, Irvine, CA) with only minimal regurgitation after the procedure. One month after the mitral valve intervention, long episodes of third-degree AV-block with repeated episodes of syncope were documented.

The procedure was performed using a conventional technique for LP implantation. After right transfemoral venous access (6F) was achieved, and due to an extremely kinked venous system, an additional stiff guidewire (Lunderquist, Extra-Stiff) was inserted in the superior vena cava. After gradual dilatation of the puncture site, a 23F delivery sheath was inserted into the RA just after IVC connection. The sheath was aspirated and flushed. The LP delivery catheter was then inserted into the RA and the sheath was gently pulled back into the IVC. First, we targeted the TV in RAO 30° projection, then switched to LAO 30° caudal 20° projection and moved the delivery system through the valve (Video 1). To further position the LP on the higher RV septum, we returned to the RAO projection. After verification of the “goose neck”, the LP was deployed. The optimal fixation was achieved when at least two tines were engaged during the tug test. The pacing parameters were not optimal neither in the higher and lower RV septum nor in the RV apex. The fourth position in the RVOT revealed excellent pacing and sensing parameters (threshold—0.38 V/0.24 ms; sensing—6.8 mV; impedance—560 Ohm). The fixation test showed optimal engagement of three tines (Fig. 1.). The total procedure and fluoroscopy time amounted to 54 min and 6.3 min, respectively, with a dose area product (DAP) of 1191 µGy m^2^. After hemodialysis and re-compensation, the patient was discharged 5 days after the implantation. A 6-month follow-up showed stable pacing and sensing parameters (threshold—0.38 V/0.24 ms; sensing—12.6 mV; impedance—570 Ohm). Transthoracic echocardiography showed no change in pre-existing minimal paravalvular TV leakage. The RV pacing percentage was 54.6% and there was no exacerbation of heart failure symptoms. Moreover, the ejection fraction of the left ventricle improved to 24%.

**Fig. 1:**
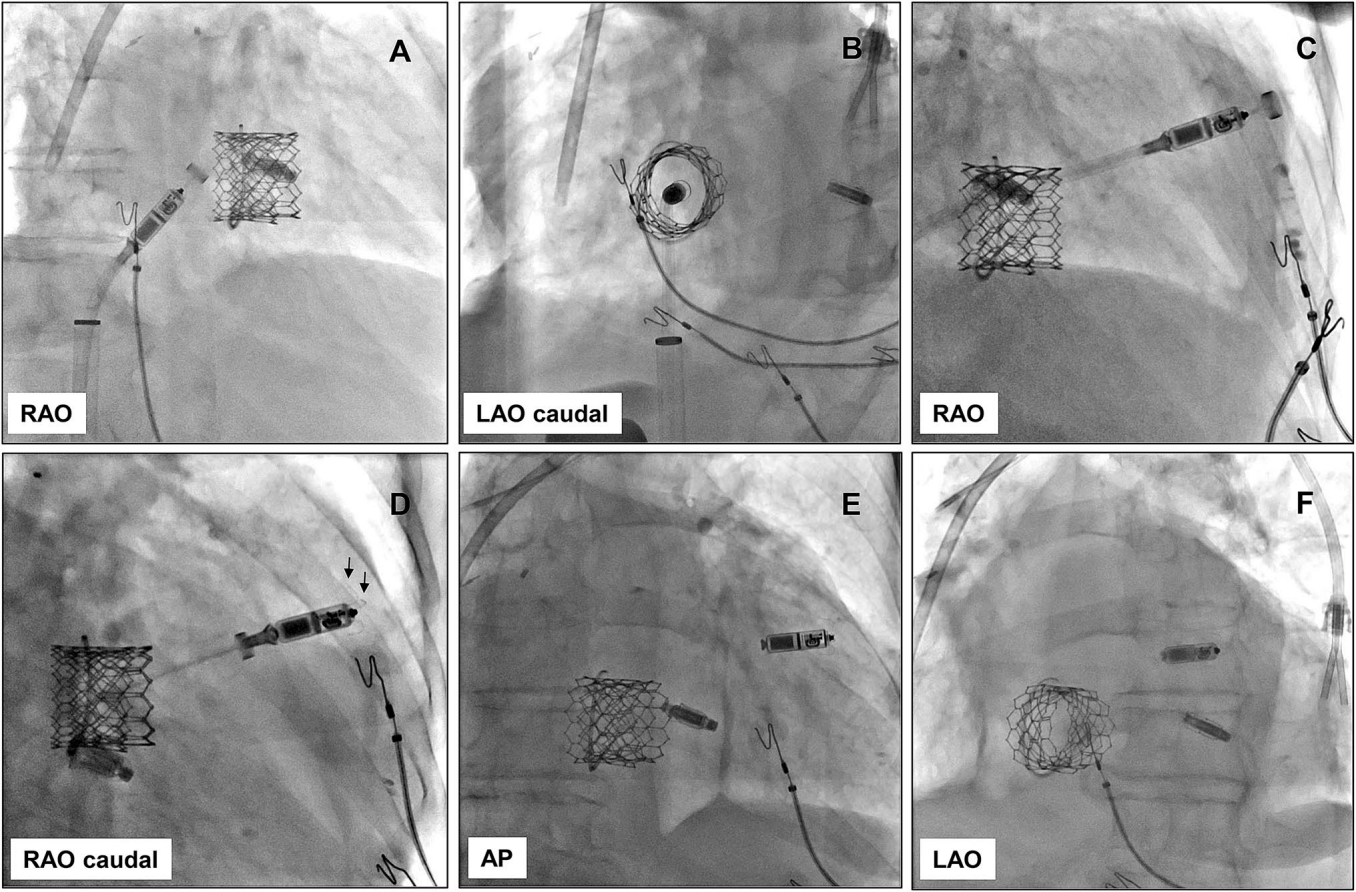
Fluoroscopic views of the leadless pacemaker implantation procedure. **A** Positioning of the LP in RAO projection. **B** LAO caudal projection for the crossing the implanted percutaneous valve. **C** RAO projection for the deployment of the LP in the RVOT position. **D** Optimal fixation with at least two tines (arrows) engagement during the tug test in RAO caudal projection. **E** End result in AP projection. **F** End result in LAO projection. *LP* leadless pacemaker, *RAO* right anterior oblique, *LAO* left anterior oblique, *RVOT* right ventricular outflow tract, *AP* anteroposterior

The number of tricuspid valve interventions increases annually. In the future, we will be faced with more multimorbid patients with different types of tricuspid valve interventions and indication for permanent pacing. As shown earlier, LP implantation results in decreased postoperative complications compared to traditional permanent pacemakers [2]. Due to the high risk of infectious complications in this patient cohort, the LP can be seen as a good alternative to conventional implanted devices. Implantation of the LP is safe after TV surgery and does not affect TV or bioprosthesis performance [3]. The first in-human LP implantation through the TAVI valve in tricuspid position has been published recently. According to the authors, the left jugular approach could have more benefits for this purpose [4]. To the best of our knowledge, there is no data about the LP implantation through the TAVI valve in tricuspid position via transfemoral access. The jugular access could be indeed more attractive if the angulation of the valve is towards the SVC as described by Hale et al. In our case, the implanted tricuspid valve was equally angulated to both the SVC and IVC. Moreover, the Demers catheter implanted via the left subclavian vein was also a limitation for the superior access in this case. Given the lack of information and experience on this topic, the main concern when performing such an intervention is a possible leaflet rupture of the bioprosthesis by a massive delivery catheter. In patients undergoing LP placement after transcatheter tricuspid intervention, transesophageal echocardiography (TEE) may be useful to allow safe passage of the introducer sheath through the valve. However, in the presented patient, the TEE procedure could have been an additional risk factor. Implantation guided with RAO—LAO caudal—RAO sequence is very helpful for the easy passage through the valve and for the avoiding the aforementioned complications. Despite that fact that four positions were tested, no influence on the valve was observed after the intervention and during follow-up.

The presented patient also has an indication for an ICD, and if such a decision is made, a subcutaneous ICD is currently the device of choice. Based on the literature and our personal experience, no interactions with the LP are expected. In the future, LP will be available from other device companies and will be compatible with implanted S-ICDs to allow pacing, ATP and defibrillation. Moreover, Friedman et al. report on a novel extravascular ICD with pacing capability, which if it becomes available could broaden the therapeutic options for patients such as the presented case [5].

Leadless pacemaker could be the preferred strategy when permanent pacing is required after tricuspid valve intervention in high-risk multimorbid patients.

### Supplementary Information

Below is the link to the electronic supplementary material.

Targeting the TV in RAO 30° projection and switching to LAO 30° caudal 20° projection for the safe passage of the delivery system through the valve (MOV 2432 KB)

## Data Availability

The data sets generated and analyzed during the current study are available from the corresponding author on reasonable request.
